# The complete mitochondrial genome of *Hymenocera picta* (Malacostraca: Decapoda: Hymenoceridae)

**DOI:** 10.1080/23802359.2018.1481779

**Published:** 2018-07-27

**Authors:** Chia-Hsuan Sung, Chen-Cheng Cheng, Jenn-Kan Lu, Liang-Jong Wang

**Affiliations:** a Planning and Information Division, Fisheries Research Institute, Keelung, Taiwan;; b Penghu Marine Biology Research Center, Fisheries Research Institute, Penghu, Taiwan;; c Department of Aquaculture, National Taiwan Ocean University, Keelung, Taiwan;; d Forest Protection Division, Taiwan Forestry Research Institute, Taipei, Taiwan

**Keywords:** Mitochondria, mitogenome, Harlequin shrimp, *Hymenocera picta*

## Abstract

In this study, the complete mitochondrial genome sequence of the *Hymenocera picta* is reported for the first time. The length of genome is 15,786 bp, including 13 protein-coding genes, two ribosomal RNA genes, and 21 transfer RNA genes. Nucleotide composition of the whole mitogenome was 37.26% A, 28.42% T, 21.92% C, and 12.40% G. The AT and GC skewness of mitogenome sequence was 0.135 and 0.277, respectively. The reconstructed phylogenetic relationships of 22 Decapoda species based on 13 protein-coding genes were highly supported and the clade of all Palaemonoidea shrimps included had a high support value. Our results shall provide a better understanding in the evolutionary histories of the Decapoda.

Harlequin shrimp, *Hymenocera picta* was first described by Dana in 1852. This species has circum-tropical distribution, throughout the Indo-Pacific and distributed from the Red Sea, eastern Africa, to Indonesia and northern Australia, central and eastern Pacific (Prakash and Kumar 2013).

The most particular characteristic of the species is the unique coloured blotches of body, and the morphology of the chela. The Harlequin shrimp is territorial and normally live in pairs (Wickler 1973). The female is generally larger than males and shows the obvious colouration in the ventral region of the abdominal segments. Males and females are sexually dimorphic and form monogamous pair bond, the adults are not known to change the sex (Fiedler 2002). Harlequin Shrimp shows very special feeding behaviour, exclusively feeds on starfish. In reef tank, the Harlequin is a very popular marine ornamental species, because of the unique and attractive coloured pattern of the body. However, the over catching led to the trend of natural population reducing. The development of the aquaculture techniques can provide the shrimp for market and reduce the ecological damage (Cheng et al. 2010).

In this study, a total of 6.3Gb next-generation sequencing paired-end reads were used to assemble the complete mitogenome sequence of the *Hymenocera picta*. The CLC Genomics Workbench (QIAGEN) was used for sequence quality analysis, data trimming, and *de novo* assembling. The locations of the protein-coding genes, ribosomal RNAs (rRNAs), and transfer RNAs (tRNAs) were predicted by using MITOS Web Server (Bernt et al. 2013) and identified by alignment with other mitogenome of Decapoda shrimps. The AT and CG skew was calculated according to the following formulas: AT skew= (A – T)/(A + T) and AT skew= (C – G)/(C + G) (Perna and Kocher 1995). The phylogenetic analysis was done by using MEGA6 (Tamura et. al. 2013).

The complete mitogenome of *H. picta* is 15,786 bp in length (GenBank Accession No. MF804409), including 13 protein-coding genes, two rRNA genes, and 21 tRNA genes. The total nucleotide composition of the *H. picta* mitogenome was 37.26% for A, 28.42% for T, 21.92% for C, and 12.0% for G. The AT and GC skewness of mitogenome sequence was 0.135 and 0.277, showing the A-skew and C-skew. The skew statistic of protein coding gene was –0.168 and 0.035, showing the T-skew and the same C-skew. We reconstructed the phylogenetic relationships of 22 Decapoda species based on 13 protein-coding genes with Maximum Likelihood (ML) criteria by using MEGA6. Bootstrap values (1000 replications) greater than 70% are shown at the branch nodes (Figure 1). The majority of nodes had support values higher than 70% and 11 were 100% supported. *H. picta* was grouped with all Palaemonoidea shrimps included and the clade of Palaemonoidea was highly supported (87–100%). Our results shall provide a better understanding in the evolutionary histories of the Decapoda.

**Figure 1.**
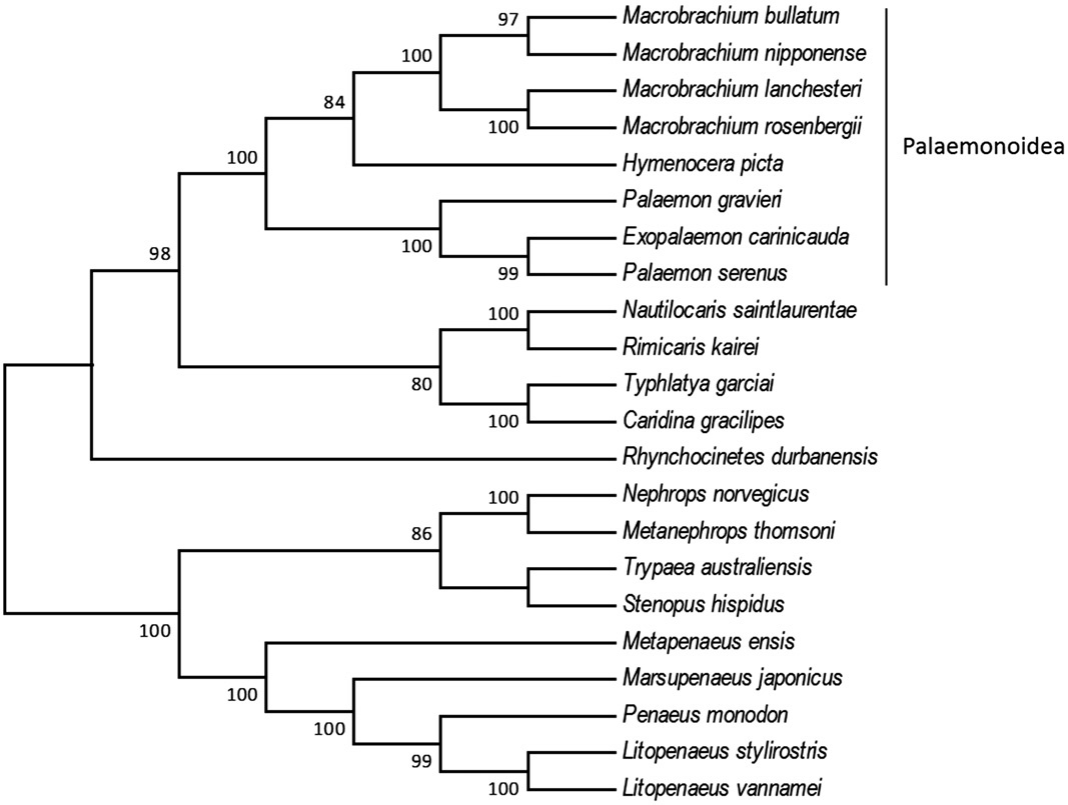
Phylogenetic tree of the 22 Decapoda species based on the sequence of 13 protein-coding genes. The tree was reconstructed with the Maximum Likelihood (ML) criteria using MEGA v.6 (Tamura et al. 2013). Bootstrap values (1000 replications) greater than 70% are shown at the branch nodes. *Macrobrachium bullatum* (KM978918), *Macrobrachium nipponense* (HQ830201), *Macrobrachium lanchesteri* (NC_012217), *Macrobrachium rosenbergii* (AY659990), *Hymenocera picta* (MF804409), *Palaemon gravieri* (NC_029240), *Exopalaemon carinicauda* (EF560650), *Palaemon serenus* (NC_027601), *Nautilocaris saintlaurentae* (NC_021971), *Rimicaris kairei* (NC_020310), *Typhlatya garciai* (KX844720), *Caridina gracilipes* (NC_024751), *Rhynchocinetes durbanensis* (NC_029372), *Nephrops norvegicus* (LN681403), *Metanephrops thomsoni* (NC_027608), *Trypaea australiensis* (NC_026225), *Stenopus hispidus* (NC_018097), *Metapenaeus ensis* (NC_026834), *Marsupenaeus japonicas* (AP006346), *Penaeus monodon* (AF217843), *Litopenaeus stylirostris* (EU517503), *Litopenaeus vannamei* (KT596762).
